# First Isolation of *Leishmania* from Northern Thailand: Case Report, Identification as *Leishmania martiniquensis* and Phylogenetic Position within the *Leishmania enriettii* Complex

**DOI:** 10.1371/journal.pntd.0003339

**Published:** 2014-12-04

**Authors:** Thatawan Pothirat, Adisak Tantiworawit, Romanee Chaiwarith, Narissara Jariyapan, Anchalee Wannasan, Padet Siriyasatien, Khuanchai Supparatpinyo, Michelle D. Bates, Godwin Kwakye-Nuako, Paul A. Bates

**Affiliations:** 1 Department of Medicine, Faculty of Medicine, Chiang Mai University, Chiang Mai, Thailand; 2 Department of Parasitology, Faculty of Medicine, Chiang Mai University, Chiang Mai, Thailand; 3 Department of Parasitology, Faculty of Medicine, Chulalongkorn University, Bangkok, Thailand; 4 Research Institute for Health Sciences, Chiang Mai University, Chiang Mai, Thailand; 5 Division of Biomedical and Life Sciences, Faculty of Health and Medicine, Lancaster University, Lancaster, United Kingdom; Charité University Medicine Berlin, Germany

## Abstract

Since 1996, there have been several case reports of autochthonous visceral leishmaniasis in Thailand. Here we report a case in a 52-year-old Thai male from northern Thailand, who presented with subacute fever, huge splenomegaly and pancytopenia. Bone marrow aspiration revealed numerous amastigotes within macrophages. Isolation of *Leishmania* LSCM1 into culture and DNA sequence analysis (ribosomal RNA ITS-1 and large subunit of RNA polymerase II) revealed the parasites to be members of the *Leishmania enriettii* complex, and apparently identical to *L. martiniquensis* previously reported from the Caribbean island of Martinique. This is the first report of visceral leishmaniasis caused by *L. martiniquensis* from the region. Moreover, the majority of parasites previously identified as “*L. siamensis*” also appear to be *L. martiniquensis*.

## Author Summary

Visceral leishmaniasis is a potentially life threatening parasitic disease that is found in many tropical and subtropical regions of the world. We report a case of visceral leishmaniasis in northern Thailand caused by a recently described new species of *Leishmania* parasite, *Leishmania martiniquensis*. By comparing DNA sequences from this parasite with those of other isolates, we show that this parasite is more widely spread than previously thought, with several other examples in Thailand, and possibly from Europe and the USA, as well as the original location in the Caribbean island of Martinique. This parasite appears to be an emerging pathogen, which, because it causes a potentially fatal disease, needs to be investigated in detail so that its transmission can be understood and control measures can be developed.

## Introduction

The leishmaniases are a group of human parasitic diseases caused by several species of the genus *Leishmania* and transmitted by the bites of female phlebotomine sand flies [1], [2]. Their clinical presentation is highly variable but ranges from relatively benign localised cutaneous leishmaniasis, through a number of more destructive cutaneous forms including mucocutaneous leishmaniasis, to systemic visceral leishmaniasis, which can be fatal if left untreated [1]. Each species of *Leishmania* tends to cause one, but occasionally more, type of clinical disease.

Leishmaniasis occurs in many tropical and subtropical regions of the world, but until relatively recently, excluding imported cases, had not been reported from South-East Asia [2]. The first known autochthonous case was discovered in Thailand in 1996, the patient presenting with visceral leishmaniasis (VL), and with no known immunodeficiency or other underlying disease, however, the species was not identified [3]. Since then, in total 13 cases of leishmaniasis have been reported from Thailand [3]–[10], the majority of these presenting as VL with no known accompanying immunodeficiency, but also some cases of HIV co-infection with broader symptomology, and in some cases cutaneous lesions, have been described [7], [9], [10]. Although the number of cases is still relatively small this certainly underestimates the true incidence, but the clinical picture that is emerging is broadly consistent with VL as found in other endemic regions of the world. Identification of the parasites responsible for leishmaniasis in Thailand has been performed on five occasions using molecular methods [5]–[7], [9], [10]. In one instance the parasite was identified as *L. infantum* [6], a known agent of VL, albeit in a new location that requires further confirmation. However, in three of the other reports [5], [7], [10] the parasites had very similar but non-identical rRNA ITS-1 sequences that differed from other known *Leishmania* species, and the fourth [9] reported an 18S rRNA sequence identical to that of one of the earlier reports [5]. These four reports have been taken by some to indicate that one new species is responsible for leishmaniasis in Thailand, and this has been referred to in the literature as “*Leishmania siamensis*”, the first recorded usage of this name appearing in Muller *et al.* 2009 [11]. However, to date the species has not been formally named and described, and so here is referred to as “*L. siamensis*”, nor has its relationship to other species of human-infective or non-pathogenic species been established. Also the view that these reports refer to one species only, the proposed “*L. siamensis*”, needs to be treated with caution as the only common sequence analysed is the rRNA ITS-1, and it is doubtful that this sequence alone is sufficiently taxonomically reliable or informative to reach such a conclusion. Interestingly, on the basis of ITS-1 sequences reports of parasites apparently similar to “*L. siamensis*” from Europe [11], [12] and the USA [13] in horses and cows have been made, but unfortunately none of these have been isolated into culture yet to enable more detailed characterisation. Nevertheless, these reports do indicate that “*L. siamensis*” or related species are emerging pathogens that have a potentially wide geographical range.

In this report we describe the isolation for the first time of *Leishmania* from a person living in northern Thailand, and which we refer to as *Leishmania* strain Chiang Mai 1 (LSCM1). Moreover, based on large subunit of RNA polymerase II gene sequencing we show that LSCM1 appears to be identical to a parasite previously reported from the Caribbean island of Martinique, and recently named *L. martiniquensis* [14], [15]. We explore the relationship of LSCM1 to “*L. siamensis*” and other *Leishmania* species and show that both belong to the recently proposed *L. enriettii* complex [16], which appears to represent a new subgenus of *Leishmania* containing human pathogens.

## Methods

### Ethics Statement

The patient who was the source of the parasites described in this study was admitted to Maharaj Nakorn Chiang Mai hospital due to ill health with an undiagnosed condition. All the biopsy samples and other clinical investigations performed were part of routine clinical investigative procedures to determine the nature of the illness. No samples or procedures were undertaken for research purposes only. This report does not contain any identifiable information that could be used to compromise patient confidentiality.

### Isolation of DNA from bone marrow

Bone marrow aspiration was performed on the sternum with local anaesthesia, using a sternal puncture needle and 5 ml syringe. DNA extraction was performed using a QIAamp DNA Mini Kit (Qiagen), following the manufacturer's instructions.

### PCR and DNA sequencing

PCR amplification of the rRNA ITS-1 sequence was performed with LeF/LeR primers as previously described [17]. Controls were *L. donovani* (MHOM/ET/67/HU3; LV9), *L. infantum* (MCAN/ES/98/LEM-935; JPC; M5), *L. tropica* (MHOM/IR/60/LV357), *L. major* (MHOM/IL/80/Friedlin; FV1) and “*L. siamensis*” (MHOM/TH/2010/PCM2; Trang). New sequences were generated for *L. enriettii* (MCAV/BR/45/LV90) and *L. martiniquensis* (MHOM/MQ/92/MAR1; LEM2494). Amplification of the large subunit of RNA Polymerase II was performed with several primer pairs: RPOF1/RPOR1 [16]; PolIIN5/PolIIN6 (GCACTTCATGTTGGACGACT/GTACTTGGTGCGGATCTCCT); PolIIN7/PolIIN8 (AGGAGTACAGGCTGAACGAC/TGTCGTCCACTTGCCGGA); PolIIS1/S2 (GCTACCTACAGCGCAAACTC/TCCTTCAGCAAGTACTCGAAC); and PolIIS3/PolIIS4 (TGCTGAAGGAGTACAAGCTGA/CGTCGCTCTCCATATTCGC), on DNA of LSCM1, *L. martiniquensis* (MHOM/MQ/92/MAR1; LEM2494), “*L. siamensis*” (MHOM/TH/2010/PCM2; Trang) and *L. colombiensis* (IHAR/CO/96/CL500; LEM2334). Amplification was performed with proof-reading DNA polymerase (Qiagen HotStar HiFidelity Polymerase) and products directly sequenced or cloned into pCR2.1-TOPO (Invitrogen) and sequenced using commercial services. Results were checked for quality using Chromas Lite 2.1.1 (http://technelysium.com.au/).

### Promastigote culture and cryopreservation

Promastigote cultures were initiated and maintained at 26°C in 25 cm^2^ tissue culture flasks using 5–10 ml volumes of Schneider's *Drosophila* medium supplemented with 20% (v/v) fetal bovine serum, later they were also cultured in Medium 199 supplemented with 10% (v/v) fetal bovine serum and BME vitamins. Promastigotes were cryopreserved in 7.5% (v/v) glycerol in culture medium and stored at −80°C and liquid nitrogen.

### Phylogenetic analysis

Initial alignments and analyses were performed using Clustal W2 (http://www.ebi.ac.uk/Tools/msa/clustalw2/). For phylogenetic analysis, alignment and tree building programmes in MEGA version 6 were used [18]. Accession numbers of sequences used are given in S1 Table. For ITS-1 sequences the Kimura 2-parameter model gave the best fitting model of sequence evolution and was used for tree construction using the maximum likelihood (ML) and neighbour joining (NJ) methods. For the large subunit of RNA polymerase II the Tamura-Nei model gave the best fitting model and was used for tree construction using the ML and NJ methods. Bootstrapping was performed on all trees with 1000 replicates.

## Results

### Case report

In July 2012, a 52-year-old Thai male farmer without any known underlying diseases was admitted to Maharaj Nakorn Chiang Mai hospital, a tertiary care, university hospital in Northern Thailand, following a 2-week history of low-grade fever, fatigue and 2-kg weight loss. The patient had a history of chronic smoking but had quit for 6 months prior to admission and was previously in good health. He lived in Ban Thi district, Lamphun province, which is located 30 km to the south-east of Chiang Mai and he had never been out of this area.

Physical examination revealed body temperature of 37.8°C, heart rate of 72 beats/min, blood pressure of 120/70 mmHg, and respiratory rate of 16/min. The patient had moderate pallor without jaundice. The abdomen showed huge splenomegaly, extending 12 cm below the left costal margin, with a smooth surface, firm consistency and no tenderness. There was no hepatomegaly and no palpable lymph nodes.

Laboratory investigations revealed a haemoglobin level of 7.5 g/dL, a white blood cell count of 2,560 cells/mm^3^ (neutrophils 46%, lymphocytes 34%, eosinophils 4%, basophils 1% and monocytes 15%) and platelet count of 89,000 cells/mm^3^. Peripheral blood smears showed normochromic and normocytic red blood cells, no rouleaux formation, and decreased white blood cell count with normal maturation. Blood urea nitrogen and creatinine were 18 and 1.4 mg/dL, respectively. Hypoalbuminemia (1.9 g/dL) and hypergammaglobulinemia (8.1 g/dL) were noted. HIV serology was negative.

**Figure 1:**
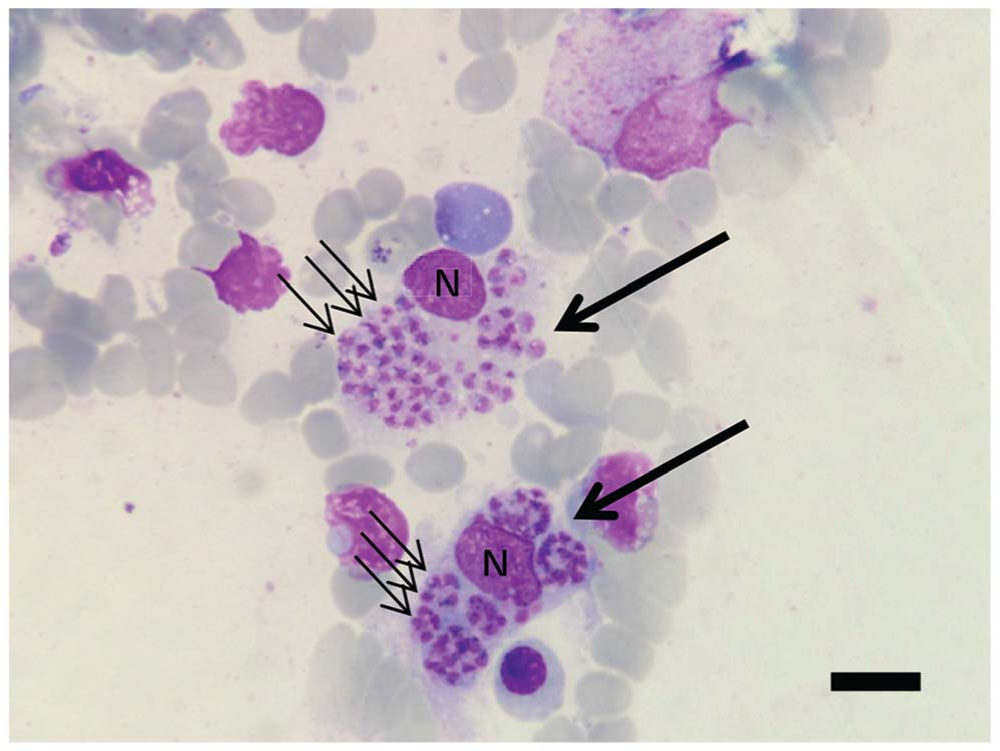
Light micrograph of *Leishmania* amastigotes in bone marrow aspirate. Two infected macrophages are stained (large arrows), each with a nucleus (N) and numerous amastigotes (examples with small arrows) within the cytoplasm. The specimen was stained using Wright's stain. The bar represents 20 µm.

**Figure 2:**
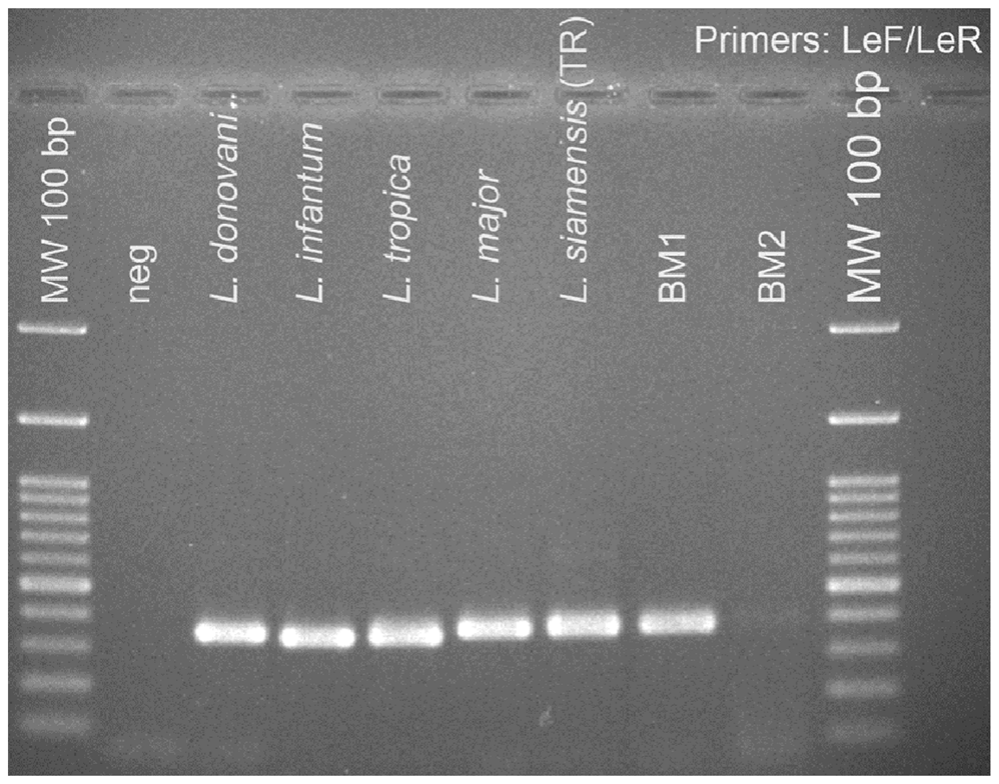
Diagnosis of leishmaniasis by PCR. Agarose gel electrophoresis showing PCR products using the LeF/LeR primers for *Leishmania* rRNA ITS-1 [17] from patient bone marrow aspirate samples compared to five *Leishmania* species, 1% gel, stained with ethidium bromide. Lane 1, negative control – no DNA; lane 2, *L. donovani*; lane 3, *L. infantum*; lane 4, *L. tropica*; lane 5, *L. major*; lane 6, “*L. siamensis*”PCM2 Trang; lane 7, BM1 is bone marrow collected 2 days after commencement of treatment; lane 8, BM2 is bone marrow collected 14 days after commencement of treatment. Size markers (MW), 100 bp ladder, are as shown.

Bone marrow (BM) aspiration showed numerous amastigotes within macrophages (Fig. 1), recognised by their size and presence of nucleus and kinetoplast. Therefore, the patient was diagnosed with visceral leishmaniasis. The BM sample was sent to the Department of Parasitology, Faculty of Medicine, Chiang Mai University for *Leishmania* species identification. Amphotericin B deoxycholate (1 mg/kg/day) was administered for 21 days, and BM samples taken on the 2^nd^ day and 14^th^ day of treatment showed decreased numbers of amastigotes. The serum creatinine became elevated to a maximum level of 3.1 mg/dL while receiving treatment. However, at 2 weeks after discharge from the hospital the patient was doing well and his serum creatinine had returned to baseline levels. DNA was extracted from BM and used as a template for PCR using the LeF/LeR primers for *Leishmania* rRNA ITS-1 [17]. A correctly sized PCR product was amplified from initial BM samples, disappearing upon treatment (Fig. 2), confirming the clinical and parasitological diagnosis.

### Parasite characterization

**Figure 3:**
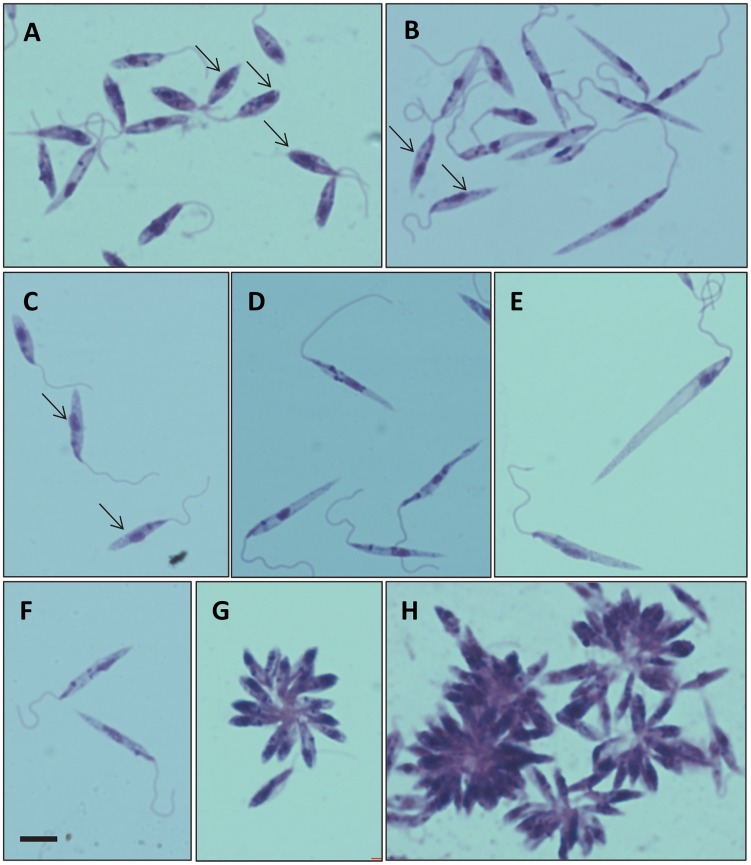
Giemsa-stained promastigote forms from culture. A–H, examples showing morphological variation of forms observed, all at the same magnification, bar in F represents 5 µm. Procyclic-like promastigotes can be observed in A (arrows);, leptomonad-like promastigotes in B and C (arrows); nectomonad-like promastigotes in D, E and F; and rosettes and aggregates in G and H.

The *Leishmania* parasites from the BM specimens were cultured in Schneider's insect medium supplemented with 20% foetal bovine serum (FBS) at 25°C. Promastigote forms were first observed on day 3. Their morphology was generally similar to that described for other *Leishmania* species (Fig. 3). A range of promastigote morphologies was observed, including some similar to procyclic promastigotes, leptomonad promastigotes and nectomonad promastigotes (Fig. 3A–F) [19]. Although free swimming individual promastigotes were readily observed, rosettes and large aggregates of promastigotes were prevalent in culture (Fig. 3G, H). The strain was sub-cultured until neither red blood cells nor white blood cells were observed and then the promastigotes were cryopreserved in liquid nitrogen. These promastigotes have been continuously maintained in this medium for over one year, and have also been successfully transferred and grown in Medium 199 supplemented with 10% FBS. The WHO code for this strain is MHOM/TH/2012/LSCM1.

### Identification and phylogenetic analysis

**Figure 4:**
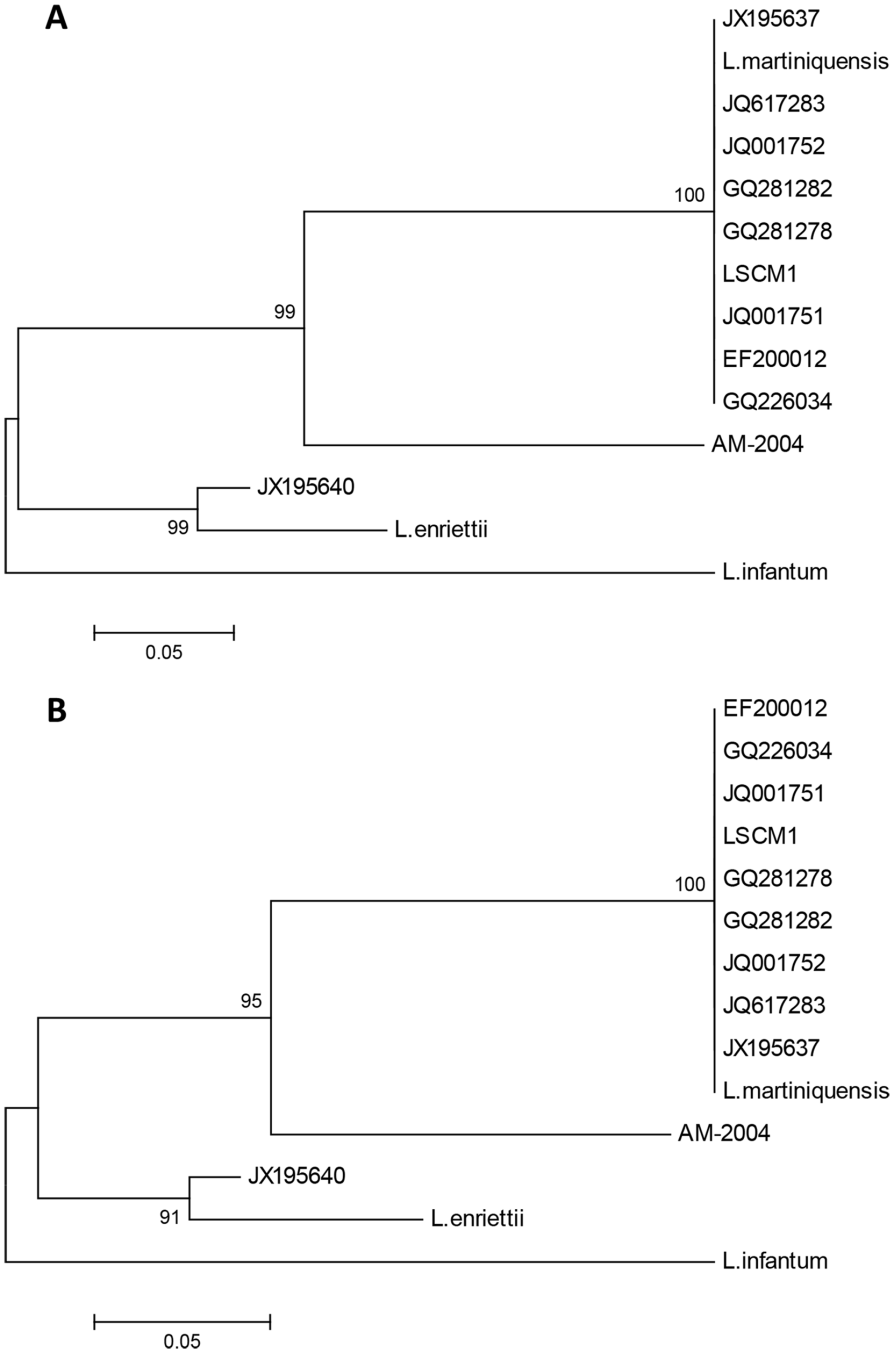
Phylogenetic analysis of *Leishmania* strain CM1 using ITS-1 sequences. A: ML tree including LSCM1 (accession number JX898938) with various “*L. siamensis*” from Thailand and elsewhere: GQ281278 is from a horse, Germany [11]; GQ281282 is from a cow, Switzerland [12]; JX195637 is of human origin from Stun, Thailand [29]; JQ617283 is from a horse, USA [13]; JQ001751, JQ001752 are human isolates from Trang and Songkhla, Thailand [10]; GQ226034 is of human origin from Chantaburi, Thailand [7]; EF200012 is of human origin from Phang-nga, Thailand [5]; JX195640 is the human PCM2 isolate from Trang, Thailand [9]. Also included are sequences from *L. enriettii*, *L. infantum*, *L. martiniquensis* and *Leishmania* from Australia (AM-2004). B: NJ tree on the same dataset. Numbers at nodes indicate bootstrap values on 1000 replicates and *L. infantum* was used as an outgroup.

An initial molecular identification of LSCM1 was performed by cloning and sequencing the PCR product of the LeF/LeR primers. This was performed on DNA extracted from both an initial BM sample taken on the first day of admission to hospital (before treatment) and on the subsequent culture derived from the BM aspirate. The resulting ITS-1 DNA sequences were identical to each other, and also were identical or very similar to several of the nucleotide sequences previously reported for “*L. siamensis*” (Figure S1A), but differed from another “*L. siamensis*”, the PCM2 Trang strain (Figure S1B). However, the LSCM1 ITS-1 sequence was also identical to that of *L. martiniquensis* (Fig. 4A, 4B). These results indicate that LSCM1 is *L. martiniquensis*, and further suggest that several other so-called “*L. siamensis*” isolates may in fact be *L. martiniquensis.* The other member of the *L. enriettii* complex shown in Fig. 4 is the parasite described by Dougall et al. [16], a *Leishmania* species from Australia infecting kangaroos that also has not yet been formally named (AM-2004).

**Figure 5:**
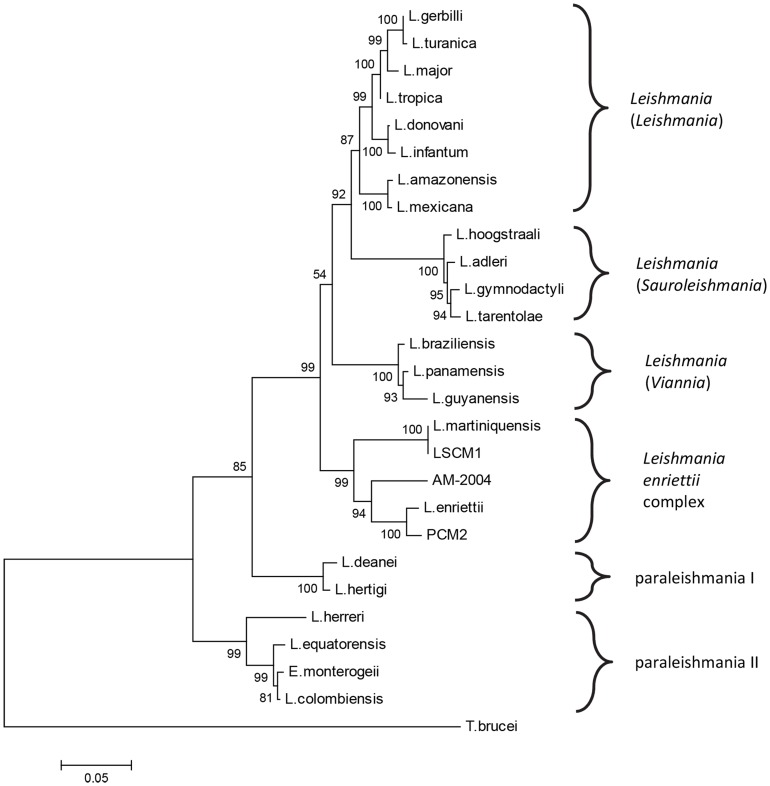
Phylogenetic analysis by ML method of *Leishmania* strain CM1 using RNA Pol II sequences. Tree with 25 species of *Leishmania* and *Endotrypanum monterogeii* using *Trypanosoma brucei* as an outgroup, based on alignment of 1191-1206 homologous nucleotide sequences. AM-2004 is *Leishmania* from Australia, PCM2 is the “*L. siamensis*” PCM2 Trang isolate. Bootstrap values from 1000 replicates are given at the nodes.

These observations required further investigation, since the ITS-1 sequence is known to be polymorphic [20] and does not contain enough informative sites to be reliable for phylogenetic analysis of *Leishmania* species. Therefore, a different target was used for this analysis, the large subunit RNA Polymerase II (Pol II) gene (*L. major* chromosome 31) that has been previously used for *Leishmania* phylogenetic analyses [14], [16], [21], [22]. LSCM1 DNA was extracted from culture and amplified with RPOF1/RPOR1 and various newly designed primer pairs (see Methods) to generate sequences of the RNA Pol II gene. Additional sequences were also generated for *L. martiniquensis*, “*L*. *siamensis*” (PCM2 Trang strain, [9]) and for *L. colombiensis*. Analysis was performed on the 25 available *Leishmania* RNA Pol II sequences (21 existing +4 new sequences from this study), *Endotrypanum monterogeii* and using *Trypanosoma brucei* as an outgroup. The resulting Maximum Likelihood (ML) tree is shown in Fig. 5. A topologically identical Neighbour Joining (NJ) tree was also generated (Figure S2). Bootstrapping provided strong support for almost all of the nodes, the only two exceptions were 54% bootstrap support for the clade including subgenera *Leishmania/Sauroleishmania/Viannia* in the ML tree (Fig. 5), however, this node received 94% support in the NJ tree; and 66% support for the clade including all species except paraleishmania II in the NJ tree (Figure S2), however, this node received 85% support in the ML tree. We also tested alternate outgroups, such as *Crithidia fasciculata*, *Leptomonas costaricensis* and *L. seymouri*, and the topologies of the resulting ML trees were similar to Fig. 5. For example, with *C. fasciculata* as an outgroup the main difference was that the paraleishmania appear as a monophyletic clade, and consequently the rooting of tree is slightly different (Figure S3).

The first point to note from the RNA Pol II analysis is that LSCM1 grouped within the *L. enriettii* complex and was identical in sequence to the RNA Pol II sequence derived for *L. martiniquensis*, in agreement with the results of ITS-1 analysis. “*L. siamensis*” PCM2 Trang also grouped within the *L. enriettii* complex, but as suggested by the ITS-1 analysis, this was clearly distinct from LSCM1/*L. martiniquensis*, being most closely related to *L. enriettii* itself. The *Leishmania* from Australia (AM-2004) occupied an intermediate position. The existence of a monophyletic clade for the *L. enriettii* complex was strongly supported by bootstrapping in both ML (Fig. 5) and NJ trees (Figure S2), and when using alternate outgroups (Figure S3), as were the positions of the individual members of the complex within the clade. Each of the subgenera *Leishmania*, *Sauroleishmania* and *Viannia* were well supported and the paraleishmania segregated into group I, with tree porcupines as primary mammalian hosts, and group II, with sloths as primary mammalian hosts.

## Discussion

The clinical features of leishmaniasis can be broadly classified into cutaneous, mucocutaneous and visceral disease. The patient described in this report had subacute fever, weight loss, pancytopenia and massive splenomegaly, all of which are common clinical features of VL [23], [24]. Although the recorded history of illness was only 2 weeks, it was apparent that the patient had endured a longer period of illness because he looked chronically ill and had hypoalbuminemia (1.9 g/dL). The incubation period of VL typically varies from 2 months to longer than a year, so the infection can be asymptomatic for a long period [1], [23].

The current first line treatments for VL consist of amphotericin B, or pentavalent antimony in areas where *Leishmania* isolates remain susceptible. Amphotericin B is available as a liposomal formulation (Ambisome), which has the advantage of lower toxicity, but amphotericin B deoxycholate can also be used if administered carefully [23], [25]. The patient was treated with amphotericin B deoxycholate at 1 mg/kg/day for 21 days. BM samples taken on the 2^nd^ and the 14^th^ day of treatment showed decreasing numbers of amastigotes by microscopy, and was clear evidence for a response to therapy. The final cure rate at 6 months of VL treated with amphotericin B deoxycholate 1 mg/kg daily for 20 days was 97% (95% CI, 95–98%) [26].

The identification and phylogenetic analysis of LSCM1 indicates that the epidemiology of leishmaniasis in Thailand is more complex than previously thought, and the limited amount of available data means that further work is clearly required to resolve questions around so-called “*L. siamensis*”. However, we can draw several conclusions from the current study. LSCM1 clearly belongs to the *L. enriettii* complex, which includes species both pathogenic (*L. martiniquensis*, “*L. siamensis*”) and non-pathogenic (*L. enriettii*, *Leishmania* from Australia) to humans, and itself appears to be an isolate of *L. martiniquensis*. *Leishmania enriettii* was discovered 70 years ago in 1944 in domestic guinea pigs from Paraná state, Brazil [27], and has only ever been isolated from these hosts in southern Brazil, mainly around Curitiba. As a species apparently non-pathogenic to human beings it has not received as much attention as other species of *Leishmania*, but has been used as a model system for chemotherapeutic and immunological studies. Its position within the genus *Leishmania* has varied with time, typically being regarded as a member of the subgenus *Leishmania* (*Leishmania*) [28]. However, more recent evidence [14], [16], and the results presented here, show that *L. enriettii* occupies a more basal position within the genus, and outside the established subgenera.

The results presented here also indicate that “*L. siamensis*” contains more than one taxon. This has been suggested by data in some previous work, namely the analyses of Leelayoova et al. [29] and Van der Auwera et al. [30]. In both of these the PCM2 Trang isolate of “*L. siamensis*” from Bualert et al. [9] appears separated from other “*L. siamensis*” isolates, a similar result to that presented here, and in Leelayoova et al. [20] PCM2 Trang was attributed to a separate lineage of “*L. siamensis*”, the TR lineage, whereas the other “*L. siamensis*” isolates were attributed to the PG lineage. However, unlike these previous analyses, here we have included three members of the *L. enriettii* complex that are distinct species, and in our analysis these are interpolated between “*L. siamensis*” PCM2 Trang and LSCM1 in both ITS-1 and RNAPolII trees, indicating the occurrence of two taxa within “*L. siamensis*”. Further, the sequence identity between LSCM1 and the recently named *L. martiniquensis* [15] indicates that the majority of what have been previously called “*L. siamensis*” in Thailand (excluding PCM2 Trang) may actually be *L. martiniquensis*, and, if true, this name should take precedence and be used for these isolates, as “*L. siamensis*” has not been formally named. Although it may seem surprising that parasites from the island of Martinique and from northern Thailand belong to the same species, *L. martiniquensis*, when the wide geographical distribution of other known putative *L. martiniquensis* is also considered (southern Thailand, Germany, Switzerland, USA) this becomes more understandable. Thus, *L. martiniquensis* appears to be a globally distributed parasite causing visceral leishmaniasis in humans but probably at low frequency. This leaves open the possibility of naming PCM2 Trang as “*L. siamensis*”, subject to formal description, with perhaps a more restricted geographical distribution. There are insufficient data currently available to fully resolve these questions, further sequencing and other characterisation needs to be done on the various isolates included within “*L. siamensis*”.

Testing of different potential outgroups produced some variations in tree topology regarding the position of the paraleishmania, whether these are a monophyletic group or not, and consequently the exact position of the root of the resulting trees. More work is clearly needed to resolve the evolutionary relationships between these groups. However, irrespective of these variations, the *L. enriettii* complex always appeared as a strongly supported monophyletic clade, with a consistent branching pattern within the clade, and was always the most basal of the clades containing human parasites.

In conclusion, this is the first report of autochthonous visceral leishmaniasis in Northern Thailand and the aetiological agent is identified as *L. martiniquensis*. Although Thailand has traditionally not been regarded as a country endemic for leishmaniasis, visceral leishmaniasis should be considered in patients presenting with subacute or prolonged fever, huge splenomegaly, and pancytopenia. Epidemiological studies are needed for prevention and control of the disease transmission in Thailand, and further work to clarify the identity and relationships of “*L. siamensis*” to other species of *Leishmania*.

## Supporting Information

Figure S1 Multiple sequence alignment of ITS-1 sequences. A: alignment of various “*L. siamensis*” from Thailand and elsewhere with LSCM1 (accession number JX898938). GQ281278 is from a horse, Germany [11]; GQ281282 is from a cow, Switzerland [12]; JX195637 is of human origin from Stun, Thailand [29]; JQ617283 is from a horse, USA [13]; JQ001751, JQ001752 are human isolates from Trang and Songkhla, Thailand [10]; GQ226034 is of human origin from Chantaburi, Thailand [7]; and EF200012 is of human origin from Phang-nga, Thailand [5]. B: alignment as in A but with the addition of JX195640, the human PCM2 isolate from Trang, Thailand [9]. Conserved sites are indicated by asterisks.
(PDF)

Figure S2 Phylogenetic analysis by NJ method of *Leishmania* strain CM1 using RNA Pol II sequences. Tree with 25 species of *Leishmania* and *Endotrypanum monterogeii* using *Trypanosoma brucei* as an outgroup, based on alignment of 1191–1206 homologous nucleotide sequences. AM-2004 is *Leishmania* from Australia, PCM2 is the “*L. siamensis*” PCM2 Trang isolate. Bootstrap values from 1000 replicates are given at the nodes.
(PDF)

Figure S3 Phylogenetic analysis by ML method of *Leishmania* strain CM1 using RNA Pol II sequences and *Crithidia fasciculata* as an outgroup. Tree with 25 species of *Leishmania* and *Endotrypanum monterogeii* using *Crithidia fasciculata* as an outgroup, based on alignment of 1191–1206 homologous nucleotide sequences. AM-2004 is *Leishmania* from Australia, PCM2 is the “*L. siamensis*” PCM2 Trang isolate. Bootstrap values from 1000 replicates are given at the nodes.
(PDF)

Table S1 Accession numbers of sequences used for phylogenetic analysis. *Those that have been generated as part of this study are indicated. †TriTrypDB identifier (http://tritrypdb.org/tritrypdb/), Stephen M. Beverley and The Genome Institute, Washington University School of Medicine.
(PDF)
