# Supplementary material for: First Isolation of Leishmania from Northern Thailand: Case Report, Identification as Leishmania martiniquensis and Phylogenetic Position within the Leishmania enriettii Complex
Source: PLoS Negl Trop Dis. 2014 Dec 4;8(12):e3339. doi: 10.1371/journal.pntd.0003339 (PMC4256172; doi:10.1371/journal.pntd.0003339)
Supplement: Figure S1 — Multiple sequence alignment of ITS-1 sequences. A: alignment of various “L. siamensis” from Thailand and elsewhere with LSCM1 (accession number JX898938). GQ281278 is from a horse, Germany [11]; GQ281282 is from a cow, Switzerland [12]; JX195637 is of human origin from Stun, Thailand [29]; JQ617283 is from a horse, USA [13]; JQ001751, JQ001752 are human isolates from Trang and Songkhla, Thailand [10]; GQ226034 is of human origin from Chantaburi, Thailand [7]; and EF200012 is of human origin from Phang-nga, Thailand [5]. B: alignment as in A but with the addition of JX195640, the human PCM2 isolate from Trang, Thailand [9]. Conserved sites are indicated by asterisks. (PDF) [file pntd.0003339.s001.pdf]

A.

```
GQ281278        ATTACACC-AAAAAACATACAGGCTAGAGAG-TAGTAGAATACATCTACTCGGGGAGGCA 58
GQ281282        ATTACACC-AAAAAACATACAGGCTAGAGAG-TAGTAGAATACATCTACTCGGGGAGGCA 58
JX195637        ATTACACC-AAAAAACATACAGG-TAGAGAG-TAGTAGAATACATCTACTCGGGGAGGCA 57
JQ617283        ATTACACC-AAAAAACATACAGG-TAGAGAG-TAGTAGAATACATCTACTCGGGGAGGCA 57
JQ001752        ATTACACC-AAAAAACATACAGG-TAGAGAG-TAGTAGAATACATCTACTCGGGGAGGCA 57
LSCM1           ATTACACC-AAAAAACATACAGG-TAGAGAG-TAGTAGAATACATCTACTCGGGGAGGCA 57
JQ001751        ATTACACC-AAAAAACATACAGG-TAGAGAG-TAGTAGAATACATCTACTCGGGGAGGCA 57
GQ226034        ATTACACC-AAAAAACATACAGG-TAGAGAG-TAGTAGAATACATCTACTCGGGGAGGCA 57
EF200012        ATTACACCCAAAAAACATACAGG-TAGAGAGGTAGTAGAATACATCTACTCGGGGAGGCA 59
                ******** ************** ******* ****************************

GQ281278        TGTTTTTTCCG-ATATGCCTTTCCCACATACACAAACACAGCAATATATATGTATATATA 117
GQ281282        TGTTTTTTCCG-ATATGCCTTTCCCACATACACAAACACAGCAATATATATGTATATATA 117
JX195637        TGTTTTTTCCG-ATATGCCTTTCCCACATACACAAACACAGCAATATATATGTATATATA 116
JQ617283        TGTTTTTTCCG-ATATGCCTTTCCCACATACACAAACACAGCAATATATATGTATATATA 116
JQ001752        TGTTTTTTCCG-ATATGCCTTTCCCACATACACAAACACAGCAATATATATGTATATATA 116
LSCM1           TGTTTTTTCCG-ATATGCCTTTCCCACATACACAAACACAGCAATATATATGTATATATA 116
JQ001751        TGTTTTTTCCG-ATATGCCTTTCCCACATACACAAACACAGCAATATATATGTATATATA 116
GQ226034        TGTTTTTTCCG-ATATGCCTTTCCCACATACACAAACACAGCAATATATATGTATATATA 116
EF200012        TGTTTTTTCCGTATATGCCTTTCCCACATACACAAACACAGCAATATATATGTATATATA 119
                *********** ************************************************

GQ281278        TACGTATATTGCTATACCCAAAAACCATACCGTAAAAAGCAAAAAGGCCGGTCGACGCCA 177
GQ281282        TACGTATATTGCTATACCCAAAAACCATACCGTAAAAAGCAAAAAGGCCGGTCGACGCCA 177
JX195637        TACGTATATTGCTATACCCAAAAACCATACCGTAAAAAGCAAAAAGGCCGGTCGACGCCA 176
JQ617283        TACGTATATTGCTATACCCAAAAACCATACCGTAAAAAGCAAAAAGGCCGGTCGACGCCA 176
JQ001752        TACGTATATTGCTATACCCAAAAACCATACCGTAAAAAGCAAAAAGGCCGGTCGACGCCA 176
LSCM1           TACGTATATTGCTATACCCAAAAACCATACCGTAAAAAGCAAAAAGGCCGGTCGACGCCA 176
JQ001751        TACGTATATTGCTATACCCAAAAACCATACCGTAAAAAGCAAAAAGGCCGGTCGACGCCA 176
GQ226034        TACGTATATTGCTATACCCAAAAACCATACCGTAAAAAGCAAAAAGGCCGGTCGACGCCA 176
EF200012        TACGTATATTGCTATACCCAAAAACCATACCGTAAAAAGCAAAAAGGCCGGTCGACGCCA 179
                ************************************************************

GQ281278        AATGCCGCGCGTATACAGTGGAAAAGTCCGTTCGTTACGGCTCTTTCTCTCTCGCGGGTG 237
GQ281282        AATGCCGCGCGTATACAGTGGAAAAGTCCGTTCGTTACGGCTCTTTCTCTCTCGCGGGTG 237
JX195637        AATGCCGCGCGTATACAGTGGAAAAGTCCGTTCGTTACGGCTCTTTCTCTCTCGCGGGTG 236
JQ617283        AATGCCGCGCGTATACAGTGGAAAAGTCCGTTCGTTACGGCTCTTTCTCTCTCGCGGGTG 236
JQ001752        AATGCCGCGCGTATACAGTGGAAAAGTCCGTTCGTTACGGCTCTTTCTCTCTCGCGGGTG 236
LSCM1           AATGCCGCGCGTATACAGTGGAAAAGTCCGTTCGTTACGGCTCTTTCTCTCTCGCGGGTG 236
JQ001751        AATGCCGCGCGTATACAGTGGAAAAGTCCGTTCGTTACGGCTCTTTCTCTCTCGCGGGTG 236
GQ226034        AATGCCGCGCGTATACAGTGGAAAAGTCCGTTCGTTACGGCTCTTTCTCTCTCGCGGGTG 236
EF200012        AATGCCGCGCGTATACAGTGGAAAAGTCCGTTCGTTACGGCTCTTTCTCTCTCGCGGGTG 239
                ************************************************************

GQ281278        TGTGTGTGGATAACGGCTCACAT 260
GQ281282        TGTGTGTGGATAACGGCTCACAT 260
JX195637        TGTGTGTGGATAACGGCTCACAT 259
JQ617283        TGTGTGTGGATAACGGCTCACAT 259
JQ001752        TGTGTGTGGATAACGGCTCACAT 259
LSCM1           TGTGTGTGGATAACGGCTCACAT 259
JQ001751        TGTGTGTGGATAACGGCTCACAT 259
GQ226034        TGTGTGTGGATAACGGCTCACAT 259
EF200012        TGTGTGTGGATAACGGCTCACAT 262
                ***********************
```

B.

```
JQ617283        ATTACACC--AAAAAACATACAGG-TAGAGAG-TAGTAGAATACATCTACTCGGGGAGGC 56
JX195637        ATTACACC--AAAAAACATACAGG-TAGAGAG-TAGTAGAATACATCTACTCGGGGAGGC 56
JQ001752        ATTACACC--AAAAAACATACAGG-TAGAGAG-TAGTAGAATACATCTACTCGGGGAGGC 56
LSCM1           ATTACACC--AAAAAACATACAGG-TAGAGAG-TAGTAGAATACATCTACTCGGGGAGGC 56
JQ001751        ATTACACC--AAAAAACATACAGG-TAGAGAG-TAGTAGAATACATCTACTCGGGGAGGC 56
GQ226034        ATTACACC--AAAAAACATACAGG-TAGAGAG-TAGTAGAATACATCTACTCGGGGAGGC 56
GQ281282        ATTACACC--AAAAAACATACAGGCTAGAGAG-TAGTAGAATACATCTACTCGGGGAGGC 57
GQ281278        ATTACACC--AAAAAACATACAGGCTAGAGAG-TAGTAGAATACATCTACTCGGGGAGGC 57
EF200012        ATTACACCC-AAAAAACATACAGG-TAGAGAGGTAGTAGAATACATCTACTCGGGGAGGC 58
JX195640        ATTACACCAAAAAAAACATACAA--TGAAAACACGGGGAGGTGTATCT-CTC-------- 49
                ********  ************   *  * *    *     *  **** ***

JQ617283        ATGTTTTTTCCG-ATA-TGCCTTTCCCACATACACAAACACAGCAATATATATGTATATA 114
JX195637        ATGTTTTTTCCG-ATA-TGCCTTTCCCACATACACAAACACAGCAATATATATGTATATA 114
JQ001752        ATGTTTTTTCCG-ATA-TGCCTTTCCCACATACACAAACACAGCAATATATATGTATATA 114
LSCM1           ATGTTTTTTCCG-ATA-TGCCTTTCCCACATACACAAACACAGCAATATATATGTATATA 114
JQ001751        ATGTTTTTTCCG-ATA-TGCCTTTCCCACATACACAAACACAGCAATATATATGTATATA 114
GQ226034        ATGTTTTTTCCG-ATA-TGCCTTTCCCACATACACAAACACAGCAATATATATGTATATA 114
GQ281282        ATGTTTTTTCCG-ATA-TGCCTTTCCCACATACACAAACACAGCAATATATATGTATATA 115
GQ281278        ATGTTTTTTCCG-ATA-TGCCTTTCCCACATACACAAACACAGCAATATATATGTATATA 115
EF200012        ATGTTTTTTCCGTATA-TGCCTTTCCCACATACACAAACACAGCAATATATATGTATATA 117
JX195640        ---TTTTTGTCAGATAACGCCTTTCCCACATACACACACACAACAATATATATGTATATA 106
                   *****  *  ***  ****************** ***** *****************

JQ617283        TATACGTATATTGCTATACCCAAAAACCATACCGTAAAAAGCAAAA---AGGCCGGTCGA 171
JX195637        TATACGTATATTGCTATACCCAAAAACCATACCGTAAAAAGCAAAA---AGGCCGGTCGA 171
JQ001752        TATACGTATATTGCTATACCCAAAAACCATACCGTAAAAAGCAAAA---AGGCCGGTCGA 171
LSCM1           TATACGTATATTGCTATACCCAAAAACCATACCGTAAAAAGCAAAA---AGGCCGGTCGA 171
JQ001751        TATACGTATATTGCTATACCCAAAAACCATACCGTAAAAAGCAAAA---AGGCCGGTCGA 171
GQ226034        TATACGTATATTGCTATACCCAAAAACCATACCGTAAAAAGCAAAA---AGGCCGGTCGA 171
GQ281282        TATACGTATATTGCTATACCCAAAAACCATACCGTAAAAAGCAAAA---AGGCCGGTCGA 172
GQ281278        TATACGTATATTGCTATACCCAAAAACCATACCGTAAAAAGCAAAA---AGGCCGGTCGA 172
EF200012        TATACGTATATTGCTATACCCAAAAACCATACCGTAAAAAGCAAAA---AGGCCGGTCGA 174
JX195640        TGTA------TTGTTATACTCA---ATTATACAGTAAACAGAAAAACAAAGGCCGGTCGA 157
                * **      *** ***** **   *  **** ***** ** ****   ***********

JQ617283        CGC-CAAATGCCGCGCGTATA----------CAGTGGAAAAGTCCGTTCGTTACGGCTCT 220
JX195637        CGC-CAAATGCCGCGCGTATA----------CAGTGGAAAAGTCCGTTCGTTACGGCTCT 220
JQ001752        CGC-CAAATGCCGCGCGTATA----------CAGTGGAAAAGTCCGTTCGTTACGGCTCT 220
LSCM1           CGC-CAAATGCCGCGCGTATA----------CAGTGGAAAAGTCCGTTCGTTACGGCTCT 220
JQ001751        CGC-CAAATGCCGCGCGTATA----------CAGTGGAAAAGTCCGTTCGTTACGGCTCT 220
GQ226034        CGC-CAAATGCCGCGCGTATA----------CAGTGGAAAAGTCCGTTCGTTACGGCTCT 220
GQ281282        CGC-CAAATGCCGCGCGTATA----------CAGTGGAAAAGTCCGTTCGTTACGGCTCT 221
GQ281278        CGC-CAAATGCCGCGCGTATA----------CAGTGGAAAAGTCCGTTCGTTACGGCTCT 221
EF200012        CGC-CAAATGCCGCGCGTATA----------CAGTGGAAAAGTCCGTTCGTTACGGCTCT 223
JX195640        CATATAAACACCGCACGTATATATATATATACAACGAAAATGTCCCTTC-TTACGGGGGC 216
                *    ***  **** ******          **  * *** **** *** ******

JQ617283        TTCTCTCTCGCGGGTGTGTGTGTGGATAACGGCTCACAT 259
JX195637        TTCTCTCTCGCGGGTGTGTGTGTGGATAACGGCTCACAT 259
JQ001752        TTCTCTCTCGCGGGTGTGTGTGTGGATAACGGCTCACAT 259
LSCM1           TTCTCTCTCGCGGGTGTGTGTGTGGATAACGGCTCACAT 259
JQ001751        TTCTCTCTCGCGGGTGTGTGTGTGGATAACGGCTCACAT 259
GQ226034        TTCTCTCTCGCGGGTGTGTGTGTGGATAACGGCTCACAT 259
GQ281282        TTCTCTCTCGCGGGTGTGTGTGTGGATAACGGCTCACAT 260
GQ281278        TTCTCTCTCGCGGGTGTGTGTGTGGATAACGGCTCACAT 260
EF200012        TTCTCTCTCGCGGGTGTGTGTGTGGATAACGGCTCACAT 262
JX195640        TTTTCTGGCG---GTGTGT-TGTGGATAACGGCTCACAT 251
                ** ***  **   ****** *******************
```
