# Supplementary figures and images for: First Isolation of Leishmania from Northern Thailand: Case Report, Identification as Leishmania martiniquensis and Phylogenetic Position within the Leishmania enriettii Complex
Source: PLoS Negl Trop Dis. 2014 Dec 4;8(12):e3339. doi: 10.1371/journal.pntd.0003339 (PMC4256172; doi:10.1371/journal.pntd.0003339)

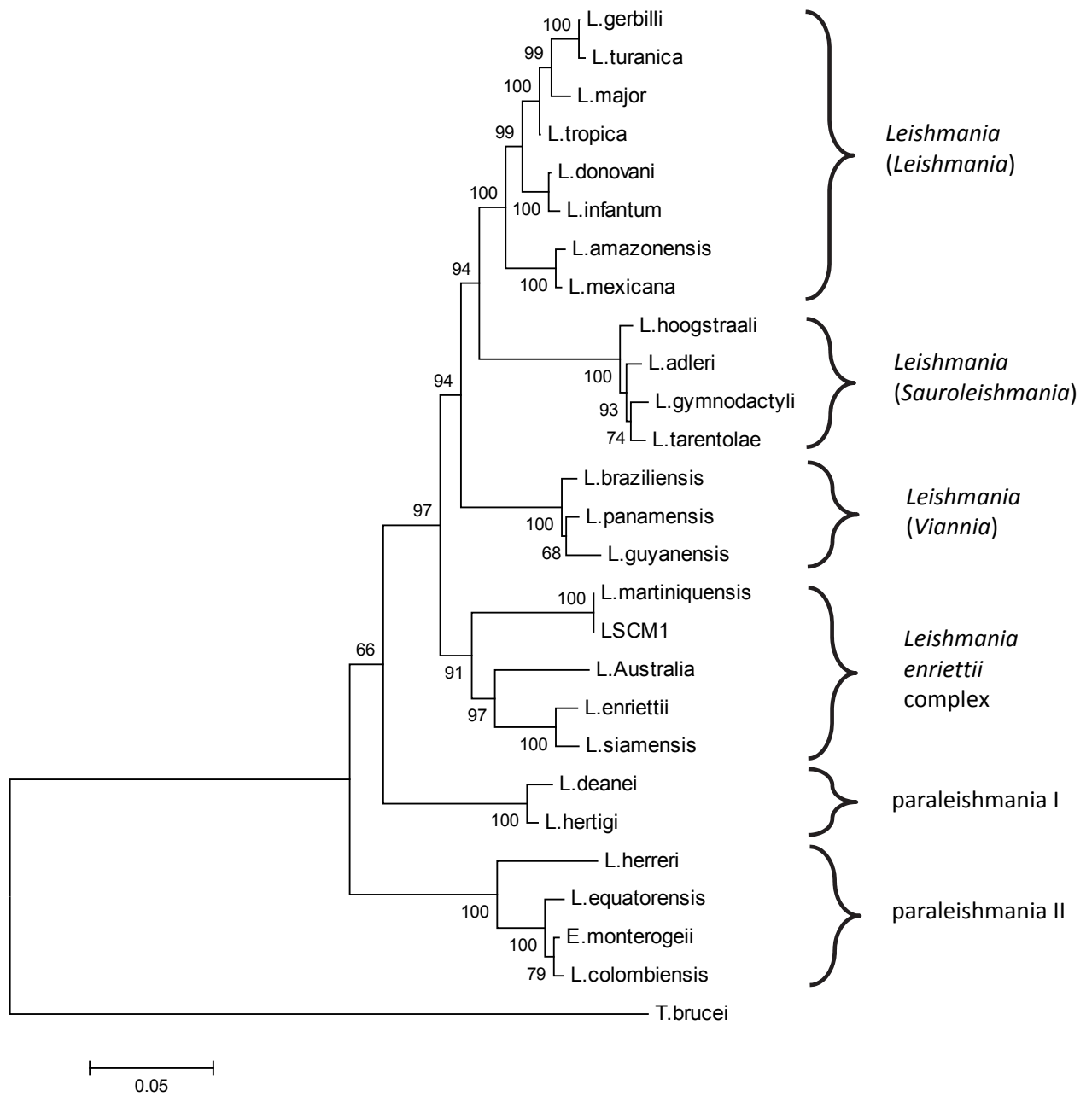

Supplement: Figure S2 — Phylogenetic analysis by NJ method of Leishmania strain CM1 using RNA Pol II sequences. Tree with 25 species of Leishmania and Endotrypanum monterogeii using Trypanosoma brucei as an outgroup, based on alignment of 1191–1206 homologous nucleotide sequences. AM-2004 is Leishmania from Australia, PCM2 is the “L. siamensis” PCM2 Trang isolate. Bootstrap values from 1000 replicates are given at the nodes. (PDF) [file pntd.0003339.s002.pdf]

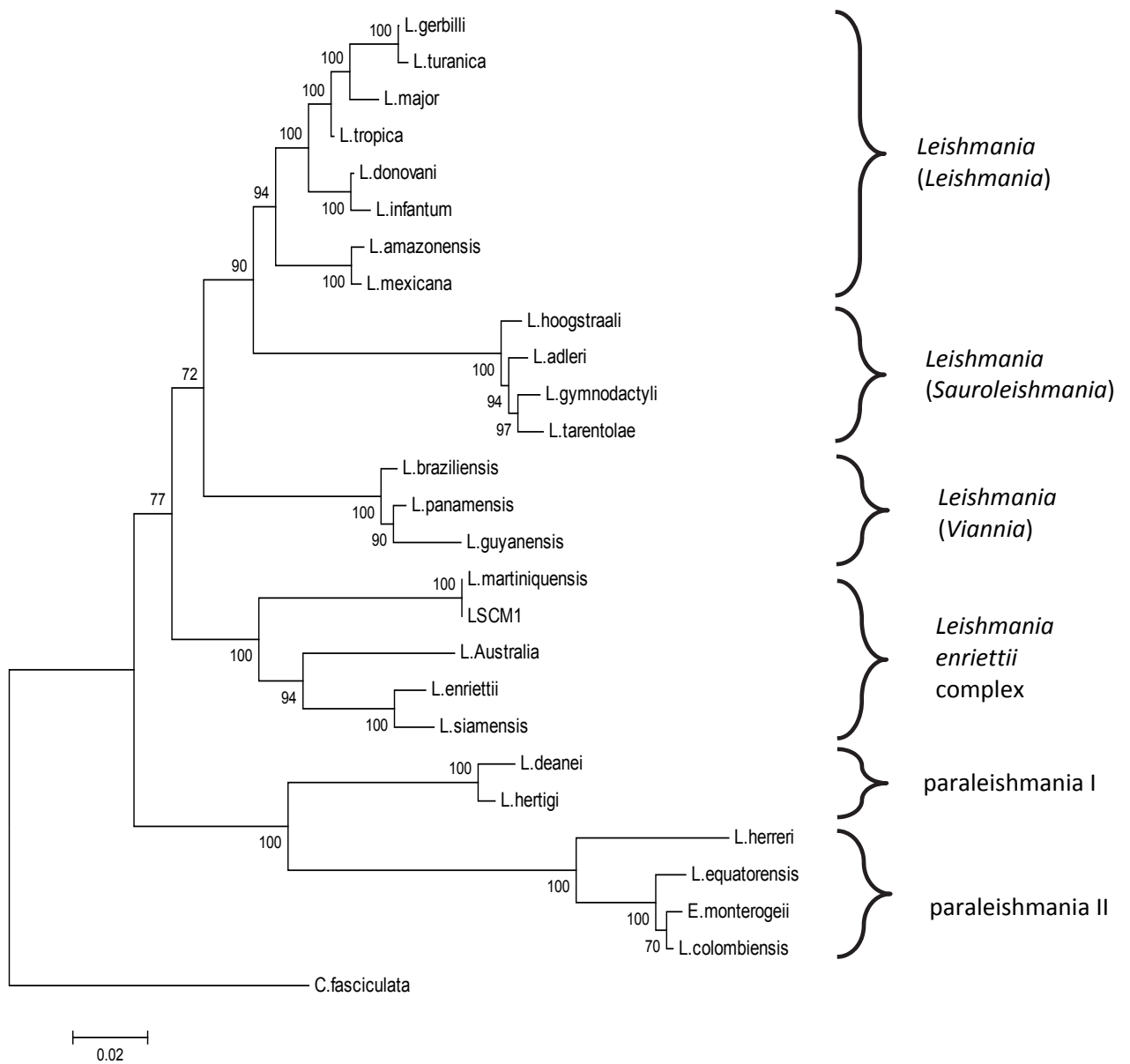

Supplement: Figure S3 — Phylogenetic analysis by ML method of Leishmania strain CM1 using RNA Pol II sequences and Crithidia fasciculata as an outgroup. Tree with 25 species of Leishmania and Endotrypanum monterogeii using Crithidia fasciculata as an outgroup, based on alignment of 1191–1206 homologous nucleotide sequences. AM-2004 is Leishmania from Australia, PCM2 is the “L. siamensis” PCM2 Trang isolate. Bootstrap values from 1000 replicates are given at the nodes. (PDF) [file pntd.0003339.s003.pdf]
