# Supplementary material for: First Isolation of Leishmania from Northern Thailand: Case Report, Identification as Leishmania martiniquensis and Phylogenetic Position within the Leishmania enriettii Complex
Source: PLoS Negl Trop Dis. 2014 Dec 4;8(12):e3339. doi: 10.1371/journal.pntd.0003339 (PMC4256172; doi:10.1371/journal.pntd.0003339)
Supplement: Table S1 — Accession numbers of sequences used for phylogenetic analysis. *Those that have been generated as part of this study are indicated. †TriTrypDB identifier (http://tritrypdb.org/tritrypdb/), Stephen M. Beverley and The Genome Institute, Washington University School of Medicine. (PDF) [file pntd.0003339.s004.pdf]

Species	WHO Code/isolate	Accession number for ITS-1
LSCM1	MHOM/TH/2012/LSCM1;Ban Thi	JX898938
"L. siamensis"	MHOM/TH/2007/PCM1; Phang-nga	EF200012
"L. siamensis"	MHOM/TH/2009/Chantaburi	GQ226034
"L. siamensis"	MHOM/TH/2010/PCM2;Trang	JX195640
"L. siamensis"	MHOM/TH/2011/CU1;Songkhla	JQ001751
"L. siamensis"	MHOM/TH/2011/Trang	JQ001752
"L. siamensis"	MEQU/GE/XXXX/Bavaria	GQ281278
"L. siamensis"	MBOS/SW/2009/Swiss	GQ281282
"L. siamensis"	MEQU/US/2011/Florida	JQ617283
"L. siamensis"	MHOM/TH/2010/PCM4;Stun	JX195637
L. martiniquensis	MHOM/MQ/1992/MAR1; LEM2494	KM677931*
Leishmania new species from Australia	MMAC/AU/2004/AM-2004;Roo1	AY495830
L. enriettii	MCAV/BR/1945/LV90	KM677932*
L. infantum	MHOM/SD/1993/452BM	AJ634371

Species	WHO Code/isolate	Accession number for RNA Polymerase II
LSCM1	MHOM/TH/2012/LSCM1;Ban Thi	KM677933*
L. adleri	RLIZ/KE/XXXX/LV30	AF009153
L. amazonensis	MHOM/BR/1973/LV78	AF009154
Leishmania new species from Australia	MMAC/AU/2004/AM-2004;Roo1	HM775497
L. braziliensis	MHOM/VE/XXXX/LBV	AF009155
L. colombiensis	IHAR/CO/1996/CL500;LEM2334	KM820662*
L. deanei	MCOE/BR/XXXX/LV402/M2909	AF009156
L. donovani	MHOM/IN/1980/DD8	AF009157
L. enriettii	MCAV/BR/1945/LV90	AF151727
L. equatorensis	MCHO/EC/1982/Lsp1;L888	DQ383655
L. gerbilli	MRHO/CN/1960/Gerbilli;LON-25	AJ304947
L. guyanensis	MHOM/SR/1987/TRUUSI	AJ304949
L. gymnodactyli	RGEC/SU/XXXX/LV247	AF009159
L. herreri	MCHO/CR/1974/LV344;CH-97	AF009160
L. hertigi	MCOE/PA/1965/C-8;LV42	AF009161
L. hoogstraali	RLIZ/SD/XXXX/LV31	AF009162
L. infantum	MCAN/ES/1998/LEM-935;JPCM5	XM_001467548
L. major	MHOM/IL/1980/Friedlin;FV1	XM_001685196
L. martiniquensis	MHOM/MQ/1992/MAR1; LEM2494	AF326982, KM820663*
L. mexicana	MNYC/BZ/1962/M379	AF009164
L. panamensis	MHOM/PA/XXXX/CIDEP004	AF009165
"L. siamensis"	MHOM/TH/2010/PCM2;Trang	JQ586202, KM820664*
L. tarentolae	RTAR/DZ/1939/LV414	AF009166
L. tropica	MHOM/SU/1958/Strain-OD	AF009167
L. turanica	IRAI/TR/1991/M87	AJ304946
Endotrypanum monterogeii	MCHO/CR/1962/LV88;A9	AF009158
Trypanosoma brucei	IsTat 1.1	J03157
Crithidia fasciculata	Cf-C1	Cf-C1_34 1,685,828-1,687,024 (minus strand)†
